# A Large Xanthomatous Hyperplastic Polyp: An Unusual Case of Dyspepsia

**DOI:** 10.7759/cureus.54083

**Published:** 2024-02-12

**Authors:** Andrea Escalante, Jeffrey H Schneider

**Affiliations:** 1 Medicine, Nova Southeastern University Dr. Kiran C. Patel College of Osteopathic Medicine, Miami, USA; 2 Gastroenterology, Broward Health Coral Springs, Coral Springs, USA

**Keywords:** bloating, xanthomatous change, gastric polyps, gastric hyperplastic polyps, dyspepsia

## Abstract

Gastric hyperplastic polyps (GHP) are one of the most common gastric epithelial polyps. They are generally asymptomatic and often discovered incidentally during endoscopic procedures. In this article, we present the case of a 36-year-old patient with dyspepsia attributed to the prolapse of a large gastric hyperplastic polyp with extensive xanthomatous change. The endoscopic findings revealed that the motion of the large polyp caused an intermittent pyloric obstruction. The large polyp was removed through a successful snare polypectomy, resolving the patient's symptoms. While dyspepsia is predominantly associated with functional causes, it is crucial to consider structural factors like GHP, particularly in the case of large polyps, as part of the differential diagnosis.

## Introduction

Gastric polyps are often discovered incidentally during esophagogastroduodenoscopy (EGD), with a reported prevalence ranging from 2% to 6% [1]. Gastric hyperplastic polyps (GHP) make up approximately 30%-93% of all cases of gastric polyps [2,3]. GHP are typically asymptomatic, though there have been reported instances in which they produce symptoms associated with intermittent gastric outlet obstruction [4]. Various risk factors have been linked to the development of GHP, including *Helicobacter pylori* infection, chronic atrophic gastritis, portal hypertension, autoimmune gastritis, prior gastric surgery, and Ménétrier disease [3]. Herein, we present a case of a healthy adult female with no past medical history who presented with episodic dyspepsia found to be secondary to a prolapsing GHP with xanthomatous changes. The incidence of GHP with xanthomatous changes remains exceedingly rare, with fewer than 10 cases reported on PubMed’s database to date [1,2].

## Case presentation

A 36-year-old female with no known past medical history presented to the gastroenterology clinic complaining of abdominal discomfort, bloating, and early satiety for several months. Symptoms were episodic, typically post-prandial, with frequent eruptions. A review of the systems was negative for heartburn, unintentional weight loss, constipation, nausea, vomiting, or diarrhea. Symptoms were refractory to a trial of antispasmodics and probiotics. The physical examination was unremarkable.

Prior workup by her primary care provider was negative for *H. pylori* infection and celiac disease. Her hematological and biochemical laboratory tests were also within normal limits. Due to persistent symptoms and lack of objective findings, an esophagogastroduodenoscopy (EGD) was recommended for further evaluation.

**Figure 1:**
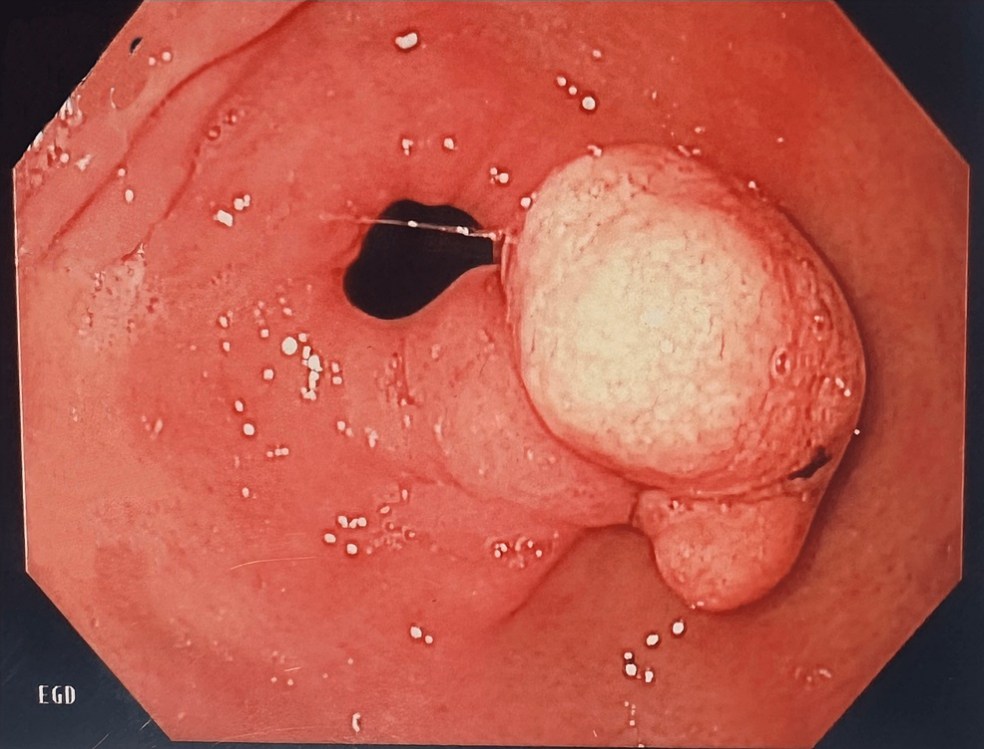
The endoscopic appearance of a 1.2 cm gastric hyperplastic polyp with a short stalk just proximal to pylorus. The yellow appearance of the polyp represents the xanthomatous changes.

During the esophagogastroduodenoscopy (EGD), a 1.2 cm pedunculated polyp with a yellow-white broad-based stalk (Figure 1) was visualized in front of the pylorus of the stomach.

**Figure 2:**
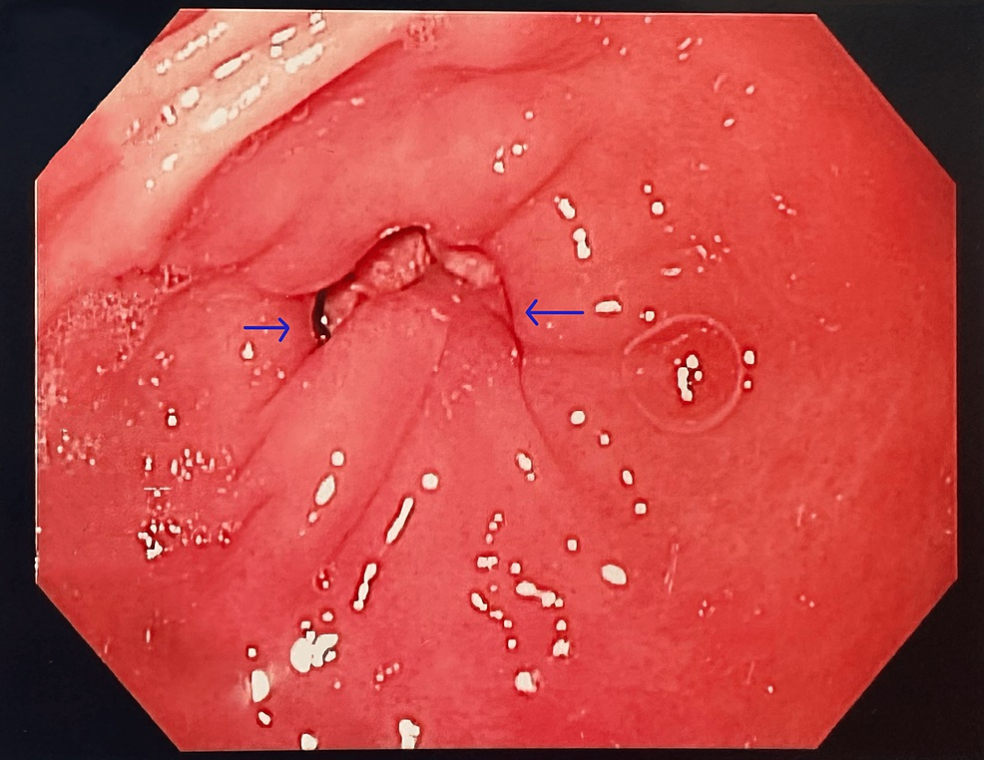
The complete prolapse of the polyp (arrows show the stalk) through the pylorus, causing transient gastric outlet obstruction

The polyp exhibited a ball-valve motion, moving in and out of the pyloric channel causing transient gastric outlet obstruction (Figure 2). No other abnormal findings were noted. Biopsies of the specimen were obtained, and histologic examinations confirmed the diagnosis of a gastric hyperplastic polyp with extensive xanthomatous change.

A few weeks after diagnostic confirmation, the polyp was endoscopically removed via snare polypectomy. The patient had an uncomplicated procedural and post-procedural course. At her follow-up appointment, she admitted to a complete resolution of her symptoms.

## Discussion

The prevalence of dyspepsia has been increasing, making it one of the primary reasons patients seek medical attention from gastroenterologists [5]. According to a review article published in the *Journal of Gastroenterology & Hepatology*, functional dyspepsia (i.e., stretching, contractions, and spasms of a structurally normal stomach causing symptoms) accounts for most cases of chronic dyspepsia [6]. In this article, we discuss a patient who required further investigation after failing first-line treatment for presumed functional dyspepsia. During upper endoscopy, an unusual structural cause of the patient’s symptoms was discovered: a large gastric hyperplastic polyp. GHP are generally asymptomatic and incidentally discovered during EGDs. However, the location, size, and pedunculated nature of this patient’s polyp were undoubtedly causing a transient mechanical obstruction in the gastric pyloric outlet.

Gastric outlet obstruction can be a consequence of a wide range of pathologies, including benign, malignant, or motility processes. Hence, symptoms of early satiety, nausea, bloating, and non-bilious vomiting are non-specific and commonly require further evaluation. In this case, complete removal by endoscopic snare polypectomy led to full resolution of the patient’s symptoms.

Unique to this case is the rare finding of a GHP with xanthomatous changes. The etiology of these changes observed in a gastric polyp remains unknown; nevertheless, current understanding suggests that such alterations may arise as a consequence of inflammatory responses or the aging process of the gastric mucosa [7]. Hyperplastic polyps account for 70%-90% of all gastric epithelial polyps [2,3]. However, the prevalence of GHP with xanthomatous changes remains exceedingly rare, with fewer than 10 cases reported on PubMed’s database to date [1,2]. In 2013, Bassullu et al. reported that out of 4497 patients who underwent upper endoscopy at their institution, only five cases (0.11%) exhibited combined lesions demonstrating features of both GHP and xanthomatous changes in three years [2]. Similarly, Fukuda et al. presented the case of a 73-year-old male with a 1 cm, pedunculated, xanthomatous GHP arising from the fornix of the stomach [8].

During our patient’s EGD, the grossly atypical nature of the GHP with yellow discoloration halted consideration for initial polypectomy as there was a concern for possible malignancy, which would require surgical rather than endoscopic removal. A benign finding of GHP with xanthomatous changes was subsequently confirmed by histology. Once malignancy was excluded, a complete polypectomy with repeat endoscopy was performed.

## Conclusions

Structural causes of dyspepsia are rare, and this case report highlights the importance of considering GHP as a potential cause of dyspepsia, especially when a large pedunculated polyp is present. While GHP are typically asymptomatic and overlooked, larger polyps with a pedunculated morphology are known risk factors for malignant transformation and may cause dyspeptic symptoms. Endoscopic removal of GHP can effectively resolve symptoms and prevent potential complications. Definitive polypectomy of a symptomatic gastric polyp leads to a satisfying outcome for both physicians and patients.

This case report demonstrates the significance of including structural causes like GHP in the differential diagnosis of symptoms of dyspepsia. Furthermore, it presents and illustrates one of the rare instances of gastric hyperplastic polyps with extensive xanthomatous change.
